# Accurate prediction of acute pancreatitis severity based on genome-wide cell free DNA methylation profiles

**DOI:** 10.1186/s13148-021-01217-z

**Published:** 2021-12-16

**Authors:** Hong-Wei Sun, Sheng-Jie Dai, Hong-Ru Kong, Jie-Xiang Fan, Fang-Yuan Yang, Ju-Qing Dai, Yue-Peng Jin, Guan-Zhen Yu, Bi-Cheng Chen, Ke-Qing Shi

**Affiliations:** 1 Department of Hepatobiliary Surgery, The First Affiliated Hospital of Wenzhou Medical University, Wenzhou, China; 2 Translational Medicine Laboratory, Key Laboratory of Intelligent Critical Care and Life Support Research of Zhejiang Province, The First Affiliated Hospital of Wenzhou Medical University, No. 1 FanHai West Road, OuHai, Wenzhou, 325000 China; 3 Key Laboratory of Intelligent Critical Care and Life Support Research of Zhejiang Province, The First Affiliated Hospital of Wenzhou Medical University, Wenzhou, China

**Keywords:** Severe acute pancreatitis, Blood markers, DNA methylation, Prediction of severity

## Abstract

**Background:**

Patients with severe acute pancreatitis (SAP) have a high mortality, thus early diagnosis and interventions are critical for improving survival. However, conventional tests are limited in acute pancreatitis (AP) stratification. We aimed to assess AP severity by integrating the informative clinical measurements with cell free DNA (cfDNA) methylation markers.

**Methods:**

One hundred and seventy-five blood samples were collected from 61 AP patients at multiple time points, plus 24 samples from healthy individuals. Genome-wide cfDNA methylation profiles of all samples were characterized with reduced representative bisulfite sequencing. Clinical blood tests covering 93 biomarkers were performed on AP patients within 24 h. SAP predication models were built based on cfDNA methylation and conventional blood biomarkers separately and in combination.

**Results:**

We identified 565 and 59 cfDNA methylation markers informative for acute pancreatitis and its severity. These markers were used to develop prediction models for AP and SAP with area under the receiver operating characteristic of 0.92 and 0.81, respectively. Twelve blood biomarkers were systematically screened for a predictor of SAP with a sensitivity of 87.5% for SAP, and a specificity of 100% in mild acute pancreatitis, significantly higher than existing blood tests. An expanded model integrating 12 conventional blood biomarkers with 59 cfDNA methylation markers further improved the SAP prediction sensitivity to 92.2%.

**Conclusions:**

These findings have demonstrated that accurate prediction of SAP by the integration of conventional and novel blood molecular markers, paving the way for early and effective SAP intervention through a non-invasive rapid diagnostic test.

**Supplementary Information:**

The online version contains supplementary material available at 10.1186/s13148-021-01217-z.

## Background

Acute pancreatitis (AP) is one of the most common gastrointestinal emergency conditions [1, 2]. Its clinical severity is stratified into three categories according to Revised Atlanta Classification (RAC): mild, moderately severe, and severe [3]. While both mild (MAP) and severe AP (SAP, including moderately severe and severe cases) patients suffer from pancreatic inflammations, SAP patients are further characterized by failure on one or more organs, and local or systemic complications. Compared to MAP, SAP patients have a much worse prognosis: on average they require significantly longer hospital stay, more frequent re-admissions, and most notably, significantly higher mortality rate [4].

Existing widely used AP severity stratification systems during the early phase of AP are either imaging-based (for example, Balthazar CT-enhanced scoring system and the computed tomography severity index (CTSI)) [5], or clinical test-based (Ranson’s score [6], the Acute Physiology and Chronic Health evaluation (APACHE-II) [7], the bedside index of severity of AP (BISAP), etc. [8]), or based on a combination of both clinical tests and patient self-reporting (pancreatic activity scoring system (PASS) [3, 9].

However, while generally useful, so far all of them have been shown to predict SAP with moderate accuracy between 0.6 to 0.8 [10, 11], some of which perform better at specificity over sensitivity in diagnoses, or vice versa [12]. Some systems, such as APACHE-II, which requires 16 tests to complete to predict AP severity, are complicated and hard to implement in typical clinical settings. Some, such as Ranson’s scores, require a minimum of 48 h after hospitalization to predict SAP, limiting the time window to initiate medical intervention [13]. Furthermore, imaging-based systems are less objective because interpretation relies on inspectors’ personal experiences [5], and enhanced CT, which is essential to identify localized pancreas complications, may actually complicate treatment by causing deterioration in pancreatic microcirculatory disturbance [14].

Given the limitations of the current AP severity prediction systems, we sought to identify novel biomarkers and establish a scoring system to accurately and objectively predict SAP during the first 24 h of hospitalization. To this end, we selected peripheral blood as the source for markers discovery: AP severity has been shown to be assessed by the levels of several different types of molecules in blood: damage-associated molecules such as HMGB1 [15], cell-free DNA [16], nucleosomes [17] and histones [18] that signal tissue damages; proinflammatory cytokines such as IL-6 [19] or IL-10 [20], which correlate with inflammation responses; levels of small molecules such as glucose, Ca^2+^, C reactive protein [21], triglycerides [22], etc. While each of these molecules may capture only one or a few aspects of the complications, organ damages or risk factors of SAP, we reasoned that integrating multiple measurements though machine learning might lead to a more accurate prediction of SAP.

Another type of biomarkers we considered was cfDNA methylation. cfDNA derives from genomic DNA released during cell death (apoptosis or necrosis), and thus carries cell-type specific epigenetic signatures from its source tissues [23]. cfDNA methylation profiles have been shown to be informative for detecting cancer in plasma, but for non-cancer diseases its clinical applicability has just begun to be explored, such as detecting acute myocardial infarction [24], type I diabetes and multiple sclerosis [23]. We reasoned that complications and organ failures that characterize SAP cause inflammatory responses, cell deaths and tissue damages, which lead to substantial releases of cfDNA species from damaged tissues that are normally at a very low level in heathy individuals’ blood, and thus generating different cfDNA methylation profiles in AP patients from healthy individuals. Moreover, MAP and SAP patients are likely to have distinct cfDNA methylation profiles due to much higher degree of complications and/or organ damages in SAP than in MAP. The signature differences in the cfDNA methylation profiles for MAP and SAP could be informative for classifying AP based on severity.

In this study, we first pursued cfDNA methylation markers that accurately classified AP or SAP in our study cohort of AP patients. We further screened conventional clinical measurements that have been performed on our study cohort and identified a subset that predicted SAP cases at an accuracy comparable to RAC. By further integrating the informative clinical measurements with cfDNA methylation markers, we derived an expanded model with a significantly improved sensitivity and overall accuracy, providing a new strategy to identify SAP cases.

## Results

### Patient characteristics and sample description

**Fig. 1:**
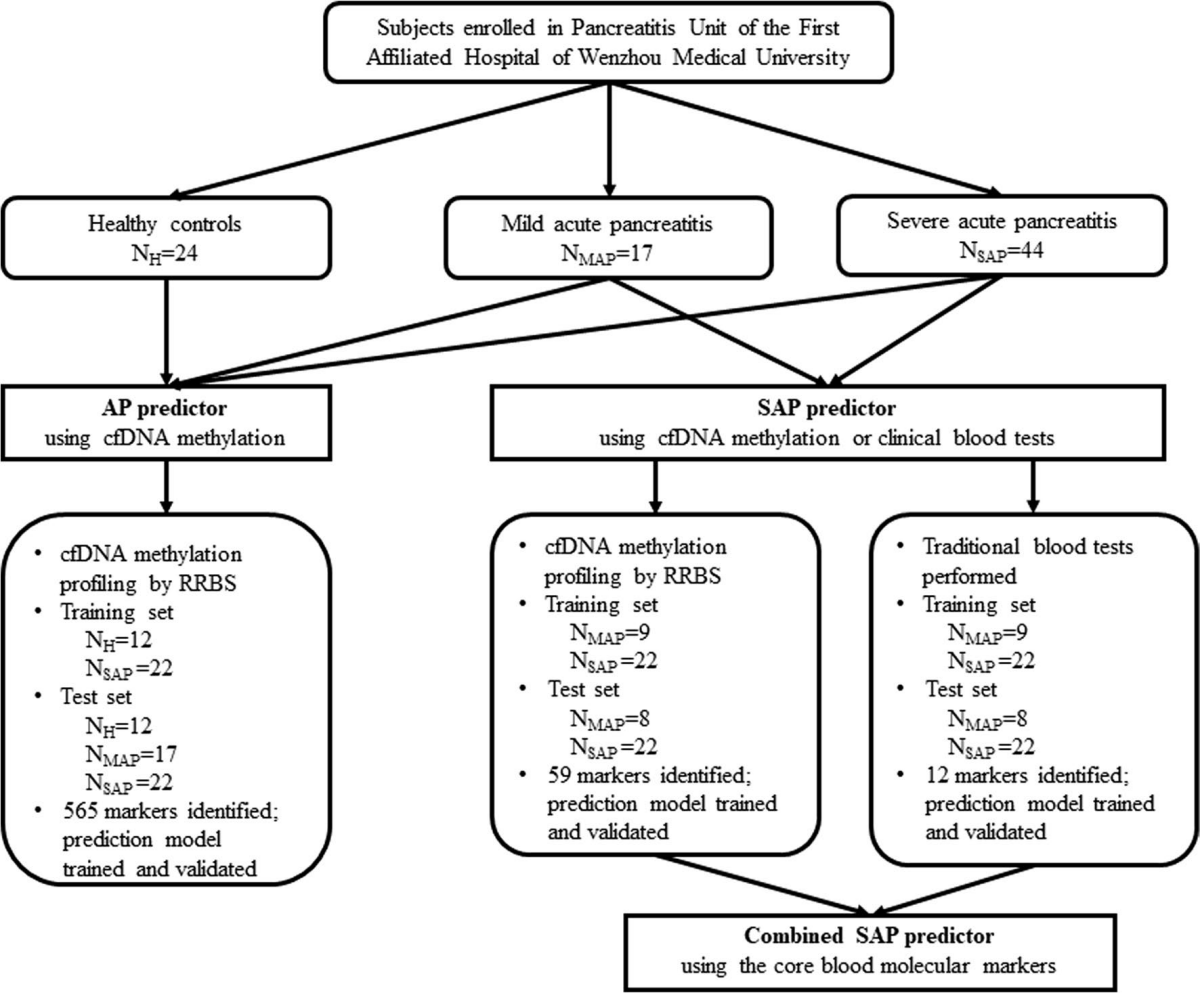
Flowchart of study design, predictive model construction and validation

Sixty-one patients diagnosed as AP were included in current study. Median age was 46.2 years (ranged from 22 to 65), and 62.3% were men. There were 17 patients with MAP, 7 with MSAP and 37 with SAP in the cohort according to the revised Atlanta classification (Fig. 1, Additional file 2: Table S1 and Table S2), respectively. For all patients, on day 1, − 3, and − 7 after hospital admission, whole blood samples were drawn to identify DNA methylation markers when AP may be rapidly advancing; additionally, patients with CT grades indicating significant pancreas pathology also had blood drawn on day 14 and − 21 to monitor the changes on methylation markers after initial treatment.

To discover tissue-specific DNA methylation markers of organs injury, we first generated genome-wide DNA methylation profiles from 120 cfDNA samples collected from multiple time points of the 45 AP patients. We also mapped DNA methylation profiles from cfDNA samples of 24 age- and sex-matched normal individuals as controls to minimize the interference of random background DNA methylation signals on AP diagnoses. cfDNA samples were extracted from both AP patients and healthy individual’s plasma samples and were prepared into DNA methylation library and sequenced on Illumina HiSeq X10 platform. The sequencing information is listed in Additional file 2: Table S3.

### Identify DNA methylation markers in plasma that detect acute pancreatitis

We reasoned that MAP and SAP may share similar DNA methylation features, but SAP samples likely have higher levels because of more damages on internal organs or tissues in SAP cases, which leads to increased release of cfDNA into blood than MAP. Therefore, we stand a better chance to identify general AP markers by first contrasting DNA methylation profiles of SAP samples with those of healthy individuals in the training phase. To improve the power of detecting subtle methylation differences in plasma DNA, we focused on a set of Methylation Haplotype Blocks (MHBs) in which local CpG methylation status are coordinated along single DNA molecules, such that tissue-specific signals are easier to detect with a haplotype-based scoring scheme [25].

To this end, we randomly assigned half of healthy control cases (12 cases) and half of SAP cases (22 cases, 69 samples) to a training set for marker discovery. The rest of the samples of either class were assigned to an independent test set for validation, as well as all MAP cases (17 cases, 39 samples). After filtering out poorly covered MHBs, a total of 43,358 MHB were used for following analysis.

**Fig. 2:**
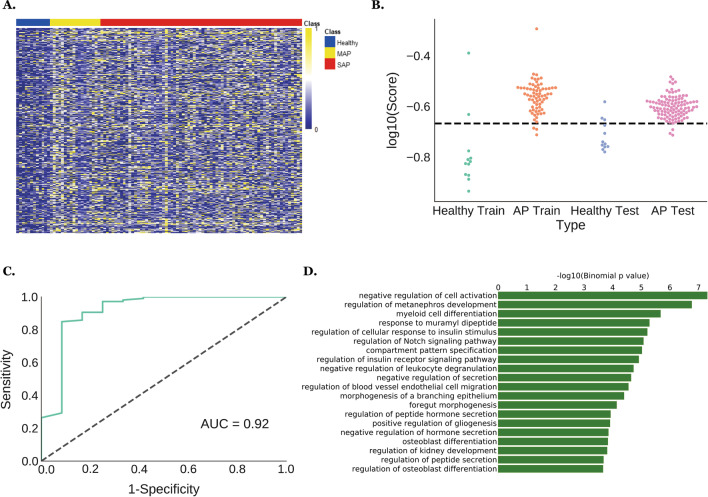
Acute pancreatitis-predicting MHBs were identified based on their uMHL scores in cfDNA samples. **A** 565 MHBs that were hypermethylated in healthy individuals’ cfDNA samples but relatively hypomethylated in APs’ samples were identified as classifiers for AP plasma. Heatmap visualizes the differences in the uMHL scores of those MHBs between healthy controls and AP samples in the training set; samples were arranged by each patient and by days; **B** swarm plot of the aggregate uMHL scores of the 565 MHB sites shows that they robustly separated healthy and AP plasma samples of either training or test set; **C** AP prediction accuracy by the (aggregated) uMHL scores of the 565 MHBs on test set AP samples over healthy controls; **D** Genes associated with the 565 identified AP markers are enriched in pancreas- and kidney-related Gene Ontology categories. AP, acute pancreatitis; cfDNA, cell free DNA; MHB, methylated haplotype block; uMHL, unmethylated haplotype load

We quantified DNA methylation patterns on MHBs using several metrics, such as methylation haploid load (MHL), average methylation frequency (AMF), etc. as classifiers for AP diagnosis [25]. Eventually we determined that uMHL, a metric that quantifies the degree and linkage disequilibrium of unmethylated CpG sites in each MHB, is the most appropriate metric to derive a classifier: indeed, we identified 565 MHBs that are hypermethylated (uMHL scores < 0.1) in over 50% of training healthy samples and also methylated to a lesser degree (uMHL scores ≥ 0.1) in more than 40% of SAP training samples (Fig. 2A, Additional file 2: Table S4). An AP-predicting model was further formulated using the aggregated uMHL scores on these MHBs to quantify each training sample. By plotting the scores of healthy and SAP samples separately, we demonstrated that these markers can accurately separate these two classes of plasma samples (*p* = 0.00085, Welch’s t-test) (Fig. 2B). The accuracy of classification was quantified using receiving operational characteristic (ROC) curve, which achieved an AUC of 0.91 (sensitivity 95.7%; specificity 83.3%) (Additional file 1: Figure S1A) on the training samples. To validate these markers, we applied the AP prediction model on the test samples using the same cutoff, 0.215 as on the training samples, and achieved accurate prediction of AP (sensitivity 97.2%; specificity 75%), confirming the robustness of our uMHL-based model in AP diagnosis (Fig. 2C).

To investigate the potential biological functions of these methylation markers, especially whether and how they are involved in the pathology of AP, we annotated these 565 MHBs using GREAT, a web portal for Gene Ontology (GO) annotation of regulatory regions [26]. We observed significant enrichments in several GO terms that are closely connected to AP pathology (Fig. 2D, Additional file 2: Table S5), including regulation of cellular response to insulin stimulus and regulation of peptide hormone secretion that are associated with normal pancreas functions; or regulation of metanephros development and foregut morphogenesis that are associated with non-pancreas organs that are often damaged during SAP (kidney and upper digestive track, respectively). We also found enrichments in genes involved in myeloid differentiation and leukocyte degranulation, which are potentially related to SAP-caused local or systematic inflammatory responses. Overall, these enriched GO categories are consistent with the known pathology of AP, especially SAP. Furthermore, we compared our AP (*N* = 565) and SAP markers (*N* = 59) with markers reported by Guo et al. [27] or the Type-I markers (tissue-specific markers) identified by Sun et al. [28] to find overlapping markers which might be tissue-specificity. Indeed, we found 46 markers by Guo et al. and 2 by Sun et al. that overlapped with our markers, respectively. The 46 markers from Guo et al. were from several organs, including pancreas, liver, lung, kidney, and GI track, all of which were known to be damaged in acute pancreatitis. The 2 overlapping markers from Sun et al. were from colon and liver, respectively. However, they did not overlap with Guo et al.’s markers. Which was not surprising considering Guo et al. and Sun et al. used different experimental methods in identifying their respective markers (NGS vs. methylation microarray) and different analytic approaches in quantifying methylation status (metrics on methylation haplotypes vs. average methylation levels on individual CpGs).

### Identify cfDNA methylation markers to classify MAP or SAP cases

We next sought to identify SAP-specific DNA methylation markers in order to assess the severity and distinguish SAP from MAP, which has an immediate clinical utility. To this end, we randomly assigned roughly half of the MAP cases (9 cases, 18 samples) and half of SAP cases (22 cases, 72 samples) to a training set for marker discovery, and the remaining cases (8 MAP cases, 21 samples, and 22 SAP cases, 64 samples) to an independent test set for validation. Based on the results from AP-specific markers discovery, we also chose uMHL as the quantitative metric for SAP marker discovery and predictive model building. MHBs were filtered based on sequencing coverage to ensure statistical robustness.

**Fig. 3:**
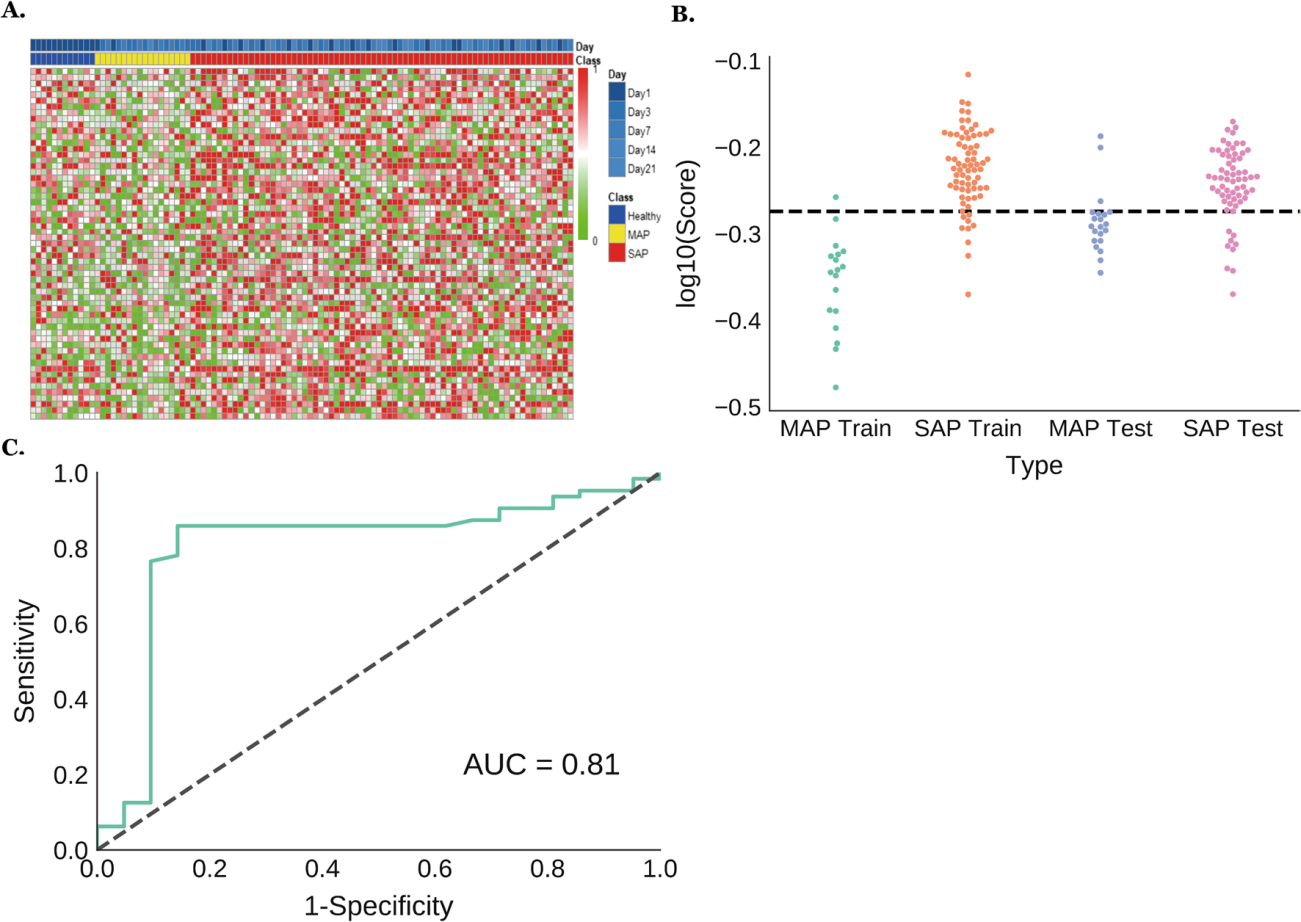
Severe acute pancreatitis-predicting MHBs were identified based on their average uMHL scores in cfDNA samples. **A** 59 MHBs whose average uMHL scores were lower in MAP samples than in SAPs’ samples were identified as SAP classifiers. Heatmap visualizes the differences in the uMHL scores of those MHBs between MAP and SAP samples in the training set; samples were arranged by each patient and by days; **B** swarm plot of the aggregate uMHL scores of the 59 MHB sites robustly separate MAP and SAP plasma samples of either training or test sample set; **C** SAP prediction accuracy by the aggregated uMHL scores of the 59 MHBs on test set samples. SAP, severe acute pancreatitis; cfDNA, cell free DNA; MAP, mild acute pancreatitis; MHB, methylated haplotype block; uMHL, unmethylated haplotype load

We performed multiple rounds of exploratory marker screenings on these MHBs and their uMHL values. Initial attempts using a single uMHL score to identify MHBs that are differentially methylated in MAP and SAP samples did not yield desired results. We then turned to an alternative strategy by looking for MHBs with mean uMHL values different between SAP and MAP samples. After evaluating multiple cutoffs for the average uMHL values and the cutoffs of maximal or minimal uMHL values, we discovered 59 MHBs, which are more methylated (max. uMHL < 0.7, mean uMHL < 0.5) in over 65% of MAP cases and less methylated (min. uMHL > 0.3, mean uMHL > 0.5) in over 65% of SAP cases, to diagnose MAP and SAP plasmas (Fig. 3A). We plotted the arithmetic average of uMHL values of these MHBs for both MAP and SAP training samples for comparison (Fig. 3B), and the results showed that SAP samples have significantly higher average uMHL scores than MAP cases (*p* = 2.83 × 10^–11^, Welch’s t-test), demonstrating that these MHBs (Additional file 2: Table S6) are less methylated in SAP samples than in MAP samples, and that the average uMHL scores can be used to differentiate MAP and SAP plasma samples. Then, 565 (SAP + MAP vs Control) and 59 (SAP vs MAP) markers were intersected, by which only 1 overlapping marker was found (PTPN1 (− 317,389), CEBPB (+ 2126)). Indeed, we used the average uMHL scores to classify MAP and SAP training samples. With a cutoff of 0.532, we were able to classify SAP with area under the receiver operating characteristic (AUC) = 0.97 (sensitivity 87.5%; specificity 94.4%) on the training samples. We then applied the MHB classifiers on the independent test samples for validation. Using the same cutoff as in the training set, we were able to classify MAP and SAP samples at an AUC of 0.81 (sensitivity 85.9%; specificity 85.7%) (Fig. 3C). Such an accuracy is comparable to the performance of several clinically used stratification systems for early assessment of AP severity, including APACHE-II, BISAP and Ranson’s score [29].

### Identify optimal clinical blood tests to predict SAP

We have demonstrated that a set of cfDNA methylation markers can predict the severity of AP at a comparable accuracy as several commonly used clinical AP stratification systems. To further improve the accuracy of predicting severity, we sought to integrate conventional biomarkers from body fluids to the cfDNA methylation-based SAP prediction model. A number of traditional biomarkers have been routinely used by clinicians to either diagnose AP (such as level of blood amylase or lipase) or have been used to monitor AP patients’ physiological conditions (blood electrolytes, etc.), inflammatory responses (levels of white blood cells, etc.) or organ damages (indicators for kidney, liver and lung functions, etc.). We tried to identify a small subset of these markers that are most indicative for SAP symptoms, which can be combined with the cfDNA methylation SAP markers to improve the overall SAP prediction accuracy. Furthermore, we aimed to select markers that can be measured during the first 24 h of AP patients’ hospitalization, in order to inform treatment decisions in a timely manner.

We surveyed 93 non-invasive clinical tests (Additional file 2: Table S1), which were performed on 61 AP cases and a total of 175 samples. Samples from all collection dates were used in the analyses, therefore they provided a comprehensive and dynamic measurement of key biomarkers to assess the temporal progresses in AP pathology and severity. The types of body fluids used in these tests included venous and arterial blood, and urine. We also included vital signs such as body temperature in our analyses. For benchmarking the performance, a RAC grade was given to each case to evaluate the prediction accuracy of SAP prediction by selected clinical tests’ results.

**Fig. 4:**
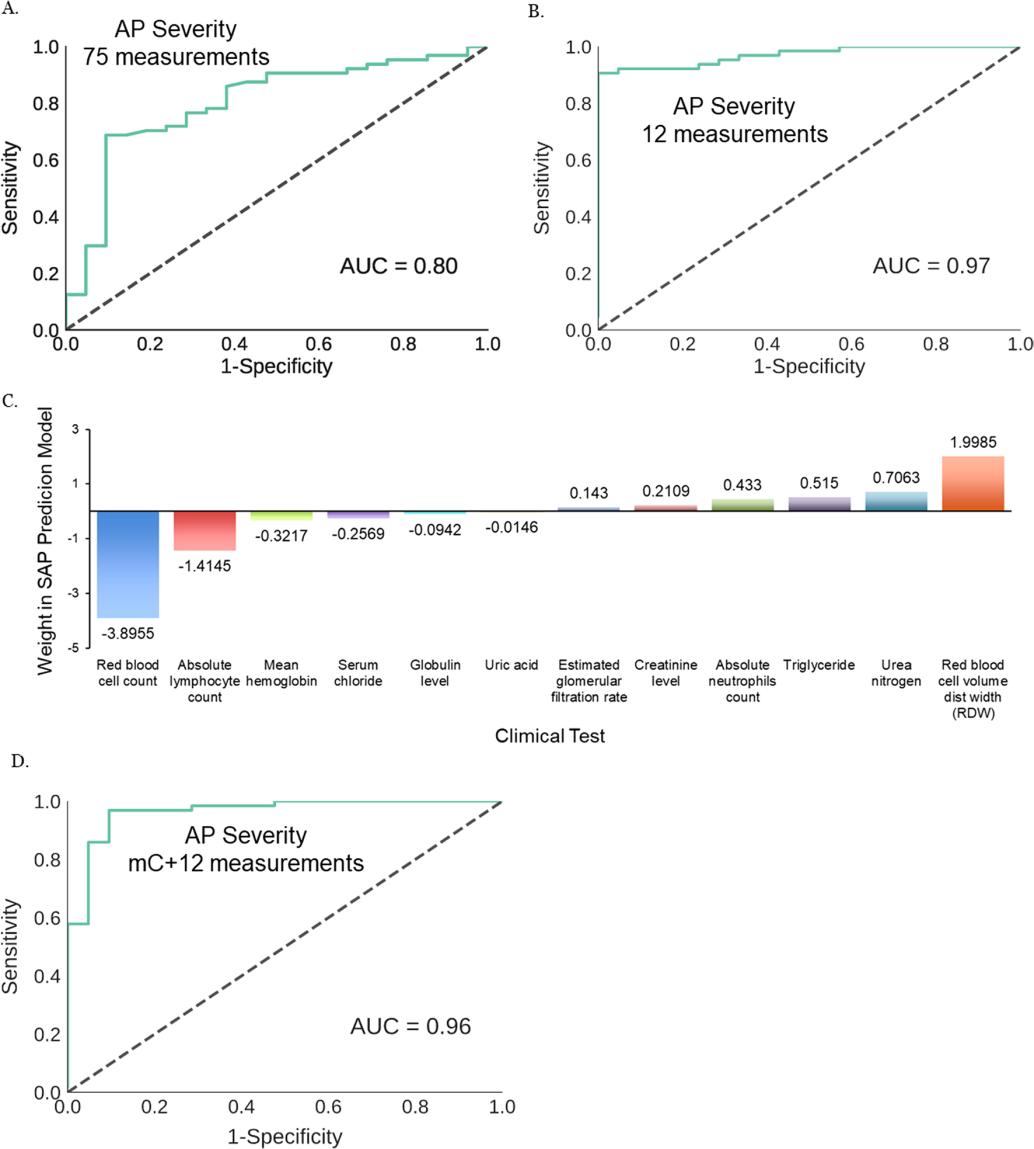
Blood levels of biomarkers measured by routine clinical tests can be used to accurately diagnosis SAP during its early stage. **A** SAP prediction accuracy by undiscriminatingly using 75 available measures from 93 body fluids-measuring clinical tests; **B** 12 venous blood-based tests identified from the training set built an SAP model that classified test set MAP and SAP samples with high accuracy; **C** members of the 12-biomarker model may either positively or negatively predict SAP. **D** When being incorporated to SAP prediction model using aggregate uMHL scores of cfDNA, the 12 venous biomarkers significantly improved its overall prediction accuracy. SAP, severe acute pancreatitis; cfDNA, cell free DNA; MAP, mild acute pancreatitis; uMHL, unmethylated haplotype load

We first performed a proof-of-principle prediction of SAP samples using all available clinical test results. We trained the all-markers model using a training set (9 MAP cases, 18 samples; 22 SAP cases, 72 samples), and detected SAP cases at a reasonable level of accuracy (AUC = 0.8) in the test set (8 MAP cases, 21 samples; 22 SAP cases, 64 samples) (Fig. 4A). This suggested that a significant number of clinical tests in this all-tests SAP prediction model are informative of pathologies that define SAP, so even without any marker selection, the all-test model was still capable of predicting SAP with moderate accuracy. We reasoned that by removing underperforming measurements with regard to detection accuracy, we should be able to further simplify and improve the predictor to an accuracy comparable to RAC. Meanwhile the large number of tests also allowed us to choose the ones that can be completed within 24 h after the collection of body fluids, thus enable early SAP diagnosis.

We then focused on 66 tests that measure biomarkers in venous blood. This was mainly because venous blood contains the majority of measurable biomarkers, and is safe and easy to collect, and many of venous blood-based tests return results within 24 h after blood collection. Body temperature was also included due to the convenience for measurement. We filtered 66 clinical tests based on data availability. This resulted in keeping 57 tests for marker discovery. Using the Recursive Feature Elimination algorithm of python package “sklearn” and the training set samples, we screened those 57 venous blood-based tests by recursively and gradually pruning off tests that contributed the least to the accuracy of SAP diagnosis, and identified the top 20 tests that formulated an SAP prediction model with an AUC of 0.99 (Additional file 1: Figure S1B and Additional file 2: Table S1), which is nearly as high as that by RAC classification (1.0 by definition).

However, this model underperformed in the test set (ACU = 0.78) (Additional file 1: Figure S1C), possibly due to overfitting. To improve the prediction model, we proceeded by first keeping tests in the 20-test model that contribute the most to prediction accuracy and whose targets were known to be associated with the risk (for example, triglyceride level) or symptoms of AP (urea nitrogen caused by kidney damage and dysfunction, etc.). We also added 5 additional tests to the prediction model based on their clinical significances on AP. Among them globulin level represents inflammatory response, creatinine, uric acid and estimated glomerular filtration rate all indicate kidney damage and dysfunction, and serum chloride has been reported to be indicative of SAP [30].

We then rebuilt a logistic regression model (python package “statsmodels”) using the 25 tests and recursively removed least-contributing tests to SAP prediction accuracy based on performances on the training set. The final model (Additional file 2: Table S7) contains 7 tests from the original 20, and all 5 new ones. It classified MAP and SAP samples in the training set with an AUC of 0.95 (sensitivity: 95.82%; specificity: 83.33%). We proceeded to validate the 12-biomarker SAP prediction model on the validation set, which predicted SAP samples at an AUC of 0.97 (sensitivity: 87.5%; specificity: 100%) (Fig. 4B). Such an accuracy is nearly as high as that of RAC, therefore we believe this model is likely sufficient for routine clinical diagnosis of SAP during the first 24 h of AP patients’ hospitalization.

The 12-biomarker model mainly measured markers indicative of organs that are known to be frequently damaged in SAP, especially kidney (urea nitrogen, creatinine, etc.), or markers informative on inflammatory responses (levels of neutrophils, lymphocytes, erythrocytes, etc.), both categories are intimately connected to the main pathologies of SAP and may explain their capacity to collectively predict SAP. Among them two measurements on red blood cell level and volume have the highest overall weight in the prediction model (Fig. 4C), followed by markers indicative of inflammation (neutrophil and lymph levels), and then by those of kidney functions.

Finally, we built an expanded SAP prediction model by combining average uMHL scores of the predefined 59 cfDNA methylation markers with the 12 clinical tests and performed logistic regression on these markers using training set (9 MAP cases, 18 samples; 22 SAP cases, 72 samples). The expanded model (Additional file 2: Table S8) was able to classify an indepedent test set comprising of 8 MAP cases (21 samples) and 22 SAP cases (64 samples), achieving an AUC of 0.96 (sensitivity 92.2%; specificity 90.5%) (Fig. 4D). While the AUC is almost identical to that of the 12-biomarker only model, the shape of the ROC curve is slightly different, such that the sensitivity was improved from 87.5 to 92.2%. This is significant in the pancreatitis clinics, because a minor reduction of specificity from 100 to 90.5% is manageable since it does not lead to adverse outcomes. In contrast, identifying SAP more sensitively and early allows for timely adjustment of the treatment options, such as fluid resuscitation, enteral nutrition, interventional endoscopic, continuous regional arterial infusion and surgical treatments, which have been well documented for reducing the mortality of SAP patients.

## Discussion

Early detection of SAP symptoms remains a challenge in the emergency care of AP patients, and is key to SAP patients’ immediate survival and long-term prognosis. RAC, being the gold standards of AP diagnosis, requires more than 48 h to assess the severity of AP cases, which limits its utility to SAP diagnosis. Other diagnostic protocols either requires longer-than-48 h to perform, or are challenging to perform and score, hence are similarly limited in SAP diagnosis.

We approached this challenge by first identifying cfDNA methylations as molecular classifiers for AP over healthy individuals, and for SAP over MAP cases, respectively. To our knowledge, this is the first set of epigenetic markers reported for AP and SAP diagnosis. Our results showed that methylation markers for AP prediction achieved a high degree of accuracy (AUC = 0.92) that is comparable to that of RAC, and markers for SAP prediction has a sensitivity and specificity comparable to several most commonly used clinical SAP diagnosis protocols. Therefore, cfDNA methylation markers alone are at a similar level of prediction accuracy to their equivalent clinical protocols. The usage of SAP cases naturally will introduce markers derived from immune cells activated during systematic inflammatory response syndrome (SIRS), which is one of the hallmarks of SAP. Thus, it’s understandable that the most discriminating markers for AP and SAP are from cell sources, such as immune cells activated during SIRS. Moreover, it should be noted that the results indicated that most of the markers had not shown tissue-specificity according to present tissue methylation databases, though further cell type specific methylation data might help elucidate.

We further improved cfDNA methylation-based SAP prediction by adding 12 selected venous blood biomarkers to build an expanded prediction model. These markers are highly informative for systematic inflammatory responses, and/or damages on organs such as kidney. An SAP prediction model build solely based on these 12 markers has an SAP prediction accuracy (0.97) in our test cohort, nearly identical to RAC (1.0). Because the selected tests are routinely performed in the majority of hospitals, the number of tests to perform is reasonably manageable, and neither their costs nor the required volume of blood is prohibitively high, we believe that our SAP diagnostic protocols can be implemented very widely.

Practically, measuring methylation status on the set of 59 methylation markers depends on targeted methylation sequencing, which may take 2–3 days to complete. However, it is worth exploring the possibility of shortening the turnaround time to fit into a 48-h detection window: for example, gradually reducing the 59 cfDNA methylation SAP markers by the RFE algorithm may reach a point where the number of remaining markers (for example, 10 or fewer) may accommodate a PCR-based detection while simultaneously maintaining accuracy. So even when cfDNA extraction and processing steps are included, SAP detection by PCR can be completed within 48 h. Additionally, detection of cfDNA methylation markers requires performing only a single assay, instead of multiple clinical tests, to diagnose SAP, therefore it might require fewer instruments and a simpler workflow.

Our efforts on cfDNA methylation marker screening are just the beginning of identifying cfDNA signatures for AP and SAP, which in future may lead to discovering organ- and/or tissue-specific markers in cfDNA and results in molecular diagnoses of damages of specific organs.

## Conclusions

In this study, we developed a novel predictive model for AP severity based on the DNA methylation patterns of plasma DNA, a type of molecular markers that have never been explored for this clinical problem. With DNA methylation signatures alone, we demonstrated a sensitive separation of AP patients from healthy controls, as well as accurate classification of MAP versus SAP. Furthermore, using a machine learning approach, we derived an expanded model with a significantly improved sensitivity and overall accuracy by further integrating the informative clinical measurements with cfDNA methylation markers, providing a new strategy to detect clinical SAP cases.

## Methods

### Study design and participants

This study was based on a case–control design with participants randomly selected from the AP cohort organized by the Pancreatitis Unit of the First Affiliated Hospital of Wenzhou Medical University, a university-affiliated tertiary-care public hospital. The study was preformed according to Standards for the Reporting of Diagnostic Accuracy Studies guidance for observational studies. The patients diagnosed with acute pancreatitis (AP) were prospectively recruited from the First Affiliated Hospital of Wenzhou Medical University between July 2017 and November 2017. The research protocol of the study was approved by the Ethics Committee of the First Affiliated Hospital of Wenzhou Medical University (2017-136) and written informed consent was obtained from each patient or their next of kin included in the study. The study was registered in Chinese Clinical Trial Registry (ChiCTR-DDD-17012200).

AP was defined as two or more of the following conditions: characteristic abdominal pain; serum amylase and/or lipase levels three or more times the upper limit of normal; and/or an imaging study (computed tomography (CT) or magnetic resonance imaging) demonstrating changes consistent with AP. Inclusion criteria were: first episode of acute pancreatitis as defined by the revised Atlanta classification; 18 years and older; male or female; and availability of blood samples within 24 h of admission. Patients were excluded with following criteria: advanced pulmonary, cardiac, renal diseases (chronic kidney disease stage 4–5), liver cirrhosis (Child–Pugh grade B-C) or malignancy; pregnancy, chronic pancreatitis or trauma as the etiology; nonpancreatic infection or sepsis caused by a second disease; or duration of abdominal pain before admission exceeding 24 h. Twenty-four healthy volunteers matched with sex and age were included as control subjects. The severity of AP stratified as mild AP or moderately/severe AP according to revised 2012 Atlanta criteria [3]. MAP and SAP cases were randomly selected from the pool of qualified patients to match age and sex.

The primary outcome of this retrospective study was to identify the most effective predictive blood markers for SAP; The secondary outcome was to compare the new model to current existing models being run in clinics, including Ranson’s score, APACH-II and BISAP.

The demographic, clinical, and laboratory data (Additional file 2: Table S1) of all patients with AP at 1st-day, 3rd-day and 7th-day of admission was prospectively collected and maintained in an electronic database in accordance with protocol for this study, including age, sex, vital signs, physical exam findings, serum levels of aspartate transaminase, alanine transaminase, alkaline phosphatase, gamma-glutamyl transferase, total bilirubin, lactate dehydrogenase, amylase, lipase, C-reactive protein, urea nitrogen (BUN) and white blood cell, etc. The severity of AP was classified by the standard RAC protocol.

Peripheral venous blood samples were obtained from each patient and each healthy volunteer. Blood samples were transported to the clinical research center at 4 °C within 1 h. Plasma was obtained after centrifugation (3000 × g, 10 min, 4 °C) and stored at − 80 °C for further analysis.

### cfDNA methylation sequencing

Cell-free DNA from plasma samples was extracted and purified using the QIAamp Circulating Nucleic Acid kit (QIAGEN, 55114). The quality of extracted cfDNA was determined by DNA NGS 3 K Assay (PerkinElmer, CLS960013). cfDNA samples were prepared into DNA methylation libraries for reduced-representation bisulfite sequencing (RRBS): briefly, up to 20 ng ctDNA was used as input for each preparation. Input DNA was ligated to customized adaptors compatible to Illumina sequencing platform. CT conversion was performed after ligation using MethylCode Bisulfite Conversion Kit (Invitrogen, MECOV50). After purification, DNA was amplified using PfuTurbo Cx Hotstart DNA polymerase (Agilent, 600412). Libraries were purified using AMPure Beads (Beckman Coulter, A63882), pooled and size-selected using 6% TBE gels (Invitrogen, EC6265BOX). The purified library pools were quantified using the KAPA Library Quantification Kit for Illumina (Kapa Biosystems, KK4824), and were sequenced on the Illumina HiSeq × 10 platform for paired ends using 2 × 150 cycle runs. Sequencing reads were demultiplexed using the Illumina bcl2fastq Conversion Software (v2.20) and aligned to the bisulfite-converted hg19 reference genome using BWA (v0.7.12) for further downstream analyses.

### Sequencing data processing

Fastq data are trimmed by trim-galore (http://www.bioinformatics.babraham.ac.uk/projects/trim_galore/). After reads trimming, both paired-end reads were merged to a single-end reads. The single reads were mapped using Bismark-transformed hg19 genome [31] with bowtie 1 [32]. The mapped bam files were processed by in house scripts extracting the methylation haplotype information.

### Quantify DNA methylation patterns on MHB

MHBs are defined as previously described [25] using a set of whole genome bisulfite sequencing data from human tissues and cell lines. To perform quantitative analysis of the methylation patterns within individual MHBs, we calculated the Unmethylated Haplotype Load (uMHL), which is a measurement of consecutiveness of un-methylated CpGs within an MHB. Briefly, it sums the fraction of consecutively un-methlylated CpG haplotypes of each length of haplotype within an MHB.

$${\text{uMHL}} = \frac{{\mathop \sum \nolimits_{i = 1}^{l} w_{i} \times P\left( {{\text{UMH}}_{i} } \right)}}{{\mathop \sum \nolimits_{i = 1}^{l} w_{i} }}$$

*l* is the length of haplotype (the number of CpGs within an MHB). *W*_*i*_ stands for weight of each length of haplotype (we select l3 putting higher weights to longer haplotypes). P(UMH_*i*_) stands for the fraction of consecutively un-methylated haplotype of haplotypes with length i.

### Identify DNA methylation markers for AP and SAP

Candidate cfDNA methylation markers for AP diagnosis were first screened by selecting MHBs that were hypermethylated in the majority of healthy individuals but less methylated in the SAP samples of a training set. These markers and the prediction model were further validated in a test set that have both MAP and SAP samples in addition to healthy controls. The sensitivity and specificity of classifications on the test samples were calculated using the same cutoff as in the training set.

Candidate methylation markers for SAP diagnosis were first screened by identifying MHBs that, as quantified based on their uMHL scores, were hypermethylated in the majority of MAP training samples but are hypomethylated in the majority of SAP training samples. The prediction model was built by averaging uMHL scores of all identified MHBs and tested in the training set to ensure sufficient accuracy using the blood samples at 1 day after admission. It was further validated in a test set, in which the sensitivity and specificity of classifications were calculated using the same cutoff as in the training set.

### Identify clinical tests to predict SAP

Clinical tests were filtered based on availability of test results, AP cases were divided into training and test groups for model building and validation, respectively. A proof-of-principle SAP prediction model using all available biomarkers in body-fluids was built using Random Forest algorithm. For SAP models using only venous blood biomarkers, we used the Recursive Feature Elimination algorithm (python package “sklearn”) to identify a preset number of tests that classified MAP and SAP samples, based on the blood biomarkers they measured with the highest degree of accuracy in the training set. We used Python package StatsModels to build the prediction model, and validated it on test set AP samples.

We then built an expanded SAP prediction model by combining average uMHL scores of the predefined cfDNA methylation markers with the identified blood biomarkers, and performed logistic regression on the combined marker set using training set. The combined model was then tested on the validation set (Table 1).

**Table 1:** Twelve conventional tests used in the severe acute pancreatitis prediction model (SAP) prediction model

Test	Unit	Mean	Min	Max	Co-efficient
Creatinine level [33]	µmol/L	78.189	5	527	0.2109
Estimated glomerular filtration rate [34]	mL/min/1.73 m^2^	105.483	10.5	264.9	0.1430
Globulin level [35]	g/L	29.976	17.8	46.9	− 0.0942
Absolute lymphocyte count [28, 36]	10^9^/L	1.318	0.1	3.21	− 1.4145
Mean hemoglobin	pg	30.354	20.8	37.6	− 0.3217
Absolute neutrophils count [37]	× 10^9^/L	7.99	0.99	28.16	0.4330
Red blood cell distribution width [38–40]	%	13.695	11.9	24	1.9985
Red blood cell count	10^12^/L	4.174	2.13	6.06	− 3.8955
Serum chloride [30]	mmol/L	98.703	82	119	− 0.2569
Triglyceride [41, 42]	mmol/L	3.722	0.35	56.25	0.5150
Urea nitrogen [43–46]	mmol/L	6.798	1.3	40	0.7063
Uric acid	µmol/L	339.56	75	852	− 0.0146

## Supplementary Information

**Additional file 1. Figure S1:** A. cfDNA methylation markers for AP prediction achieved an AUC of 0.91 on classifying healthy and AP samples in the training set; B. The 20-marker SAP prediction model performed robustly in classifying training MAP and SAP samples; C. but it underperformed on the test set, demanding adjustment in selecting biomarkers and rebuilding prediction model.

**Additional file 2.** **Table S1.** Clinical test results for all samples. **Table S2.** Patient characteristics. **Table S3.** The sequencing information. **Table S4.** Methylation markers selected for AP classification. **Table S5.** Functional enrichments for the AP biomarkers. **Table S6.** Methylation markers selected for MAP vs SAP classification. **Table S7.** Biomarkers used in the conventional test model. **Table S8.** Weight coefficients for the SAP predictive model with 12 clinical tests and methylation score.

## Data Availability

Yes.

## Abbreviations

AP: Acute pancreatitis
MAP: Mild acute pancreatitis
SAP: Severe acute pancreatitis
RRBS: Reduced representative bisulfite sequencing
MHB: Methylated haplotype block
uMHL: Unmethylated haplotype load

## Availability of data and materials

The datasets used and/or analysed during the current study are available from the corresponding author on reasonable request.
