# Supplementary material for: Accurate prediction of acute pancreatitis severity based on genome-wide cell free DNA methylation profiles
Source: Clin Epigenetics. 2021 Dec 16;13:223. doi: 10.1186/s13148-021-01217-z (PMC8680202; doi:10.1186/s13148-021-01217-z)
Supplement: Supplementary file 1 — Additional file 1. Figure S1: A. cfDNA methylation markers for AP prediction achieved an AUC of 0.91 on classifying healthy and AP samples in the training set; B. The 20-marker SAP prediction model performed robustly in classifying training MAP and SAP samples; C. but it underperformed on the test set, demanding adjustment in selecting biomarkers and rebuilding prediction model. [file 13148_2021_1217_MOESM1_ESM.pptx]

## Slide 1
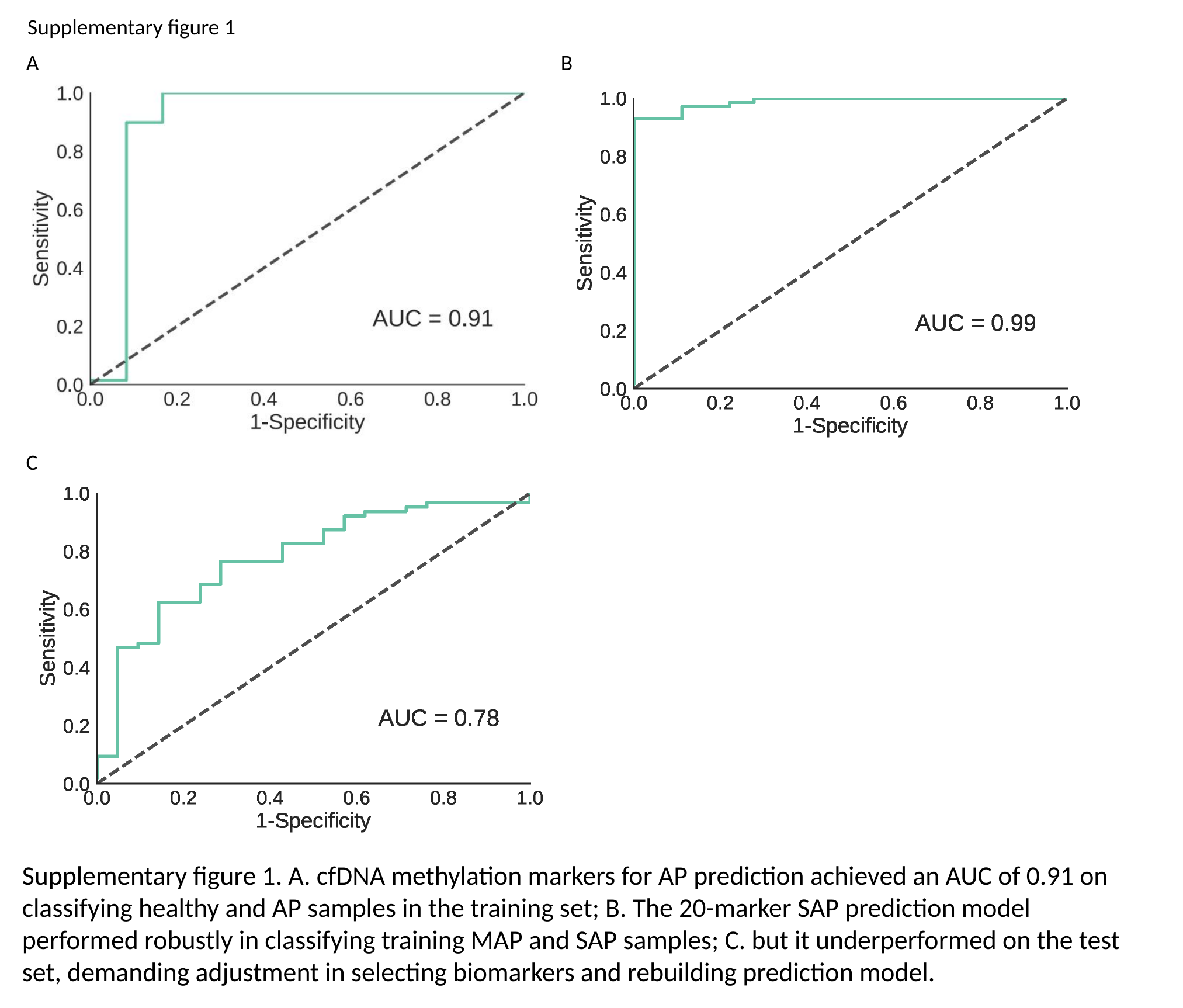

Supplementary figure 1
A
B
C
Supplementary figure 1. A. cfDNA methylation markers for AP prediction achieved an AUC of 0.91 on classifying healthy and AP samples in the training set; B. The 20-marker SAP prediction model performed robustly in classifying training MAP and SAP samples; C. but it underperformed on the test set, demanding adjustment in selecting biomarkers and rebuilding prediction model.
